# A systematic review of targeted therapy for vestibular schwannoma in patients with *NF2-*related schwannomatosis

**DOI:** 10.1093/noajnl/vdad099

**Published:** 2023-08-16

**Authors:** Shivani Chiranth, Seppo W Langer, Hans Skovgaard Poulsen, Thomas Urup

**Affiliations:** The DCCC Brain Tumor Center , Copenhagen, Denmark; University of Copenhagen, Copenhagen, Denmark; University of Copenhagen, Copenhagen, Denmark; Department of Oncology, Copenhagen University Hospital - Rigshospitalet, Copenhagen, Denmark; The DCCC Brain Tumor Center , Copenhagen, Denmark; The DCCC Brain Tumor Center , Copenhagen, Denmark; Department of Oncology, Copenhagen University Hospital - Rigshospitalet, Copenhagen, Denmark

**Keywords:** hearing loss, *NF2-*related Schwannomatosis, NF2, toxicity, Vestibular Schwannomas

## Abstract

**Background:**

One of the hallmarks of *NF2-*related Schwannomatosis *(NF2*-related SWN) is bilateral vestibular schwannomas (VS) that can cause progressive hearing impairment in patients. This systematic review was performed to investigate the efficacy and toxicity of tested targeted agents.

**Methods:**

The systematic search was conducted on PubMed and EMBASE Ovid databases from inception to October 2022, according to the Preferred Reporting Items for Systematic Reviews and Meta-Analyses (PRISMA) guidelines. The incidence of outcomes in studies involving bevacizumab and other targeted therapies was extracted. The bevacizumab results were pooled, and 95% confidence intervals (95% CI) were calculated.

**Results:**

Sixteen studies (8 prospective and 8 retrospective) testing 6 drugs were selected out of 721 search results. There were 10 studies concerning bevacizumab, with a total of 200 patients. The pooled radiographic response rate (RR) was 38% (95% CI: 31 – 45%) and the pooled hearing response rate (HR) was 45% (95% CI: 36 - 54%). The most frequent bevacizumab-related toxicities were hypertension and menorrhagia. Of other targeted therapies showing activity, lapatinib had a RR of 6% and a HR of 31%. A VEGFR vaccine showed RR in 29% and HR in 40% of patients. Both agents had a manageable safety profile.

**Conclusions:**

Bevacizumab, in comparison to other targeted agents, showed the highest efficacy. Lower dosage of bevacizumab shows comparable efficacy and may reduce toxicity. Other targeted agents, administered alone or as combination therapy, have the potential to improve outcomes for VS in patients with *NF2*-related SWN, but future clinical studies are needed.

**Key Points**

- Bevacizumab was the most effective agent with high rates of radiographic and hearing response.
- Hypertension and menorrhagia were the most common bevacizumab-related serious adverse events.
- Lower dosage of bevacizumab shows comparable efficacy and may reduce toxicity.

**Importance of the Study**

The hallmark of *NF2-*related Schwannomatosis (*NF2*-related SWN) is the development of bilateral vestibular schwannomas (VS), with hearing loss as one of the main causes of morbidity. Surgery may be necessary in symptomatic tumors but contain a high risk of iatrogenic hearing loss. In less symptomatic tumors, targeted treatment that prioritizes hearing preservation could potentially be used, instead of more invasive treatments. Here, we have conducted a systematic review of the efficacy and toxicity of various targeted treatments for VS in patients with *NF2-*related SWN. Of the 6 drugs investigated, bevacizumab had the highest efficacy, showing radiographic response and hearing improvement in 38% and 45% of patients, respectively. Hypertension and menorrhagia were the most common toxicities observed. However, we hypothesize that a lower dosage of bevacizumab might reduce toxicity. Other drugs also showed some response, but additional studies are needed to further improve the treatment of these patients.

*NF2-*related Schwannomatosis (*NF2-*related SWN) previously known as Neurofibromatosis type 2 is an autosomal dominant, tumor predisposing condition arising due to a mutation in the *NF2* tumor suppressor gene.^1–3^ The incidence at birth is approximately 1:25 000–33 000.^4^ One of the hallmarks of *NF2-*related SWN is the development of bilateral vestibular schwannomas (VS) involving the vestibular branch of the eighth cranial nerve with hearing loss as one of the main causes of morbidity.^1,2^

The current guidelines for the management of VS in *NF2-*related SWN, include observation, surgery, stereotactic radiosurgery (SRS), and targeted therapy. For each patient, the treatment modality is based on various factors like clinical presentation, tumor size, and patient preferences. In patients with large, symptomatic VS with potentially life-threatening mass effects, maximal safe surgical resection is recommended. If cranial nerve functions can be preserved, a partial resection followed by SRS can also be performed.^4^ For patients with smaller, less symptomatic tumors, there is a need for less invasive treatments that prioritize hearing preservation and tumor control.

There has been important progress in understanding the pathogenesis and molecular biology of VS in *NF2-*related SWN. Mutations in the *NF2* gene affect its protein product merlin, which in its non-mutated form inhibits growth stimulatory signaling pathways (RAS-MAPK and AKT-mTOR). Consequently, in the absence of functional merlin in VS in patients with *NF2-*related SWN, these signaling pathways are activated and cause tumor growth.^5^ Therefore, agents targeting these classical oncogenic pathways or other more unknown pathways could potentially improve the treatment of these tumors. In this systematic review, we aim to summarize the clinical efficacy and safety of different types of targeted agents for VS in *NF2-*related SWN.

## Methods

### Search Strategy

PICOS format was used to form the following search strategy^6^: NF2-related SWN patients with VS (population) treated with targeted therapy (intervention and comparator) in retrospective and prospective studies (study types) measuring radiographic tumor response and hearing response (HR) (outcomes).

The literature review was conducted according to Preferred Reporting Items for Systematic Reviews and Meta-Analyses (PRISMA) guidelines^7^ in PubMed and Ovid EMBASE databases from 2008 to October 15, 2022, using the following string: *([vestibular schwannoma] OR [acoustic neuroma]) AND ([neurofibromatosis 2] OR [neurofibromatosis type 2] OR [NF2-related Schwannomatosis]) AND ([hearing loss] OR [retrospective] OR [clinical study]).*

### Inclusion and Exclusion Criteria

The inclusion criteria were articles reporting on patients with a confirmed diagnosis of *NF2-*related SWN, not suitable for surgery or radiotherapy, treated with targeted therapy, and measuring either hearing outcome or radiographic response (RR).

The exclusion criteria were articles based on studies with under 3 patients, phase 0 or phase 1 studies, articles not in English, and articles published before 2008.

### Data Extraction

The data was extracted directly from the text, tables, and figures. The primary endpoints were radiographic tumor volume and HR as determined after completion of treatment compared to baseline values.

Radiographic tumor volume was evaluated according to the Response Evaluation in Neurofibromatosis and Schwannomatosis (ReiNS) criteria using volumetric tumor measurements of target tumor in magnetic resonance imaging. A RR was defined as > 20% decrease in tumor volume and progressive disease (PD) was defined as > 20% increase in tumor volume compared to baseline. Anything else was defined as stable disease.

HR was determined using word recognition scores (WRS) in the target ear. A 95% critical difference table was used by most studies to determine statistically significant changes in WRS.^8–10^ A HR was defined as a statistically significant increase in WRS scores and a hearing decline (HD) was defined as a statistically significant fall in WRS scores compared to baseline. Some other studies also used audiometry, speech determination scores or pure tone thresholds to measure hearing.^11–14^ However, not every patient was assessable for HR or HD. Some participants could not be evaluated for either HR or HD due to bilateral deafness or due to the presence of cochlear or brainstem implants. Moreover, only participants with WRS under 94% could be evaluated for HR and only participants with WRS over 6% could be evaluated for HD, in order for the results to be significant.

Toxicity was a secondary endpoint. Grade ≥ 3 toxicities according to the Common Toxicity Criteria for Adverse Events (CTCAE) (version 3.0 and 4.0) were evaluated.

Time to tumor progression (TTP) was not uniformly defined or measured in all studies. Most studies measured it from the date of the study drug until volumetric or hearing progression, whichever event occurred first.^15,16^

### Statistics

The individual RR, PD, HR, and HD values from the different reports were processed to find pooled values with 95% confidence intervals.

Participants were divided into 2 cohorts based on their bevacizumab dosage during the treatment (2.5 mg/kg/week vs. 5 mg/kg/week). To determine the impact of dosage on efficacy, the 2 cohorts were compared utilizing the Chi-square test (IBM SPSS statistics v. 25)

## Results

### Studies

**Figure 1.**
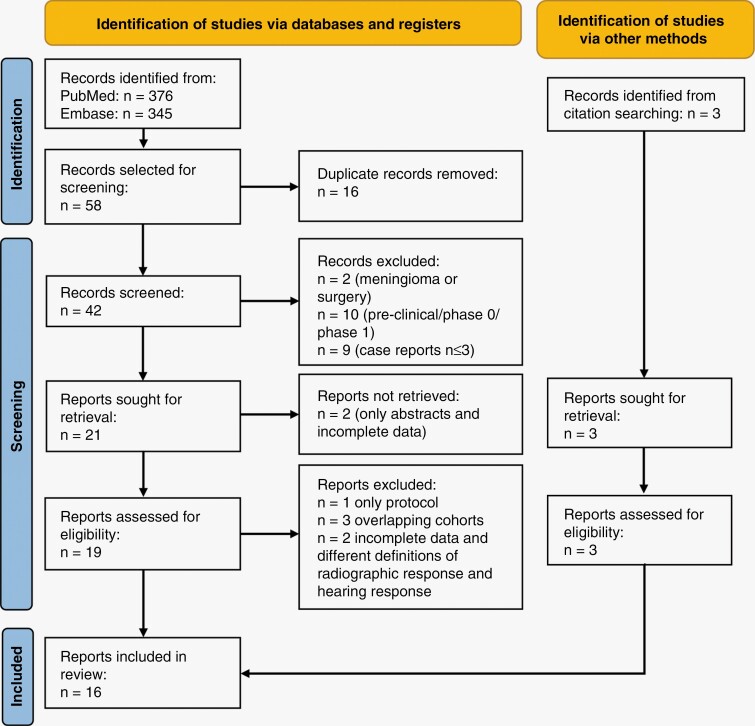
Selection process of the studies according to the Preferred Reporting Items for Systematic Reviews and Meta-Analyses (PRISMA) guidelines.

**Figure 2.**
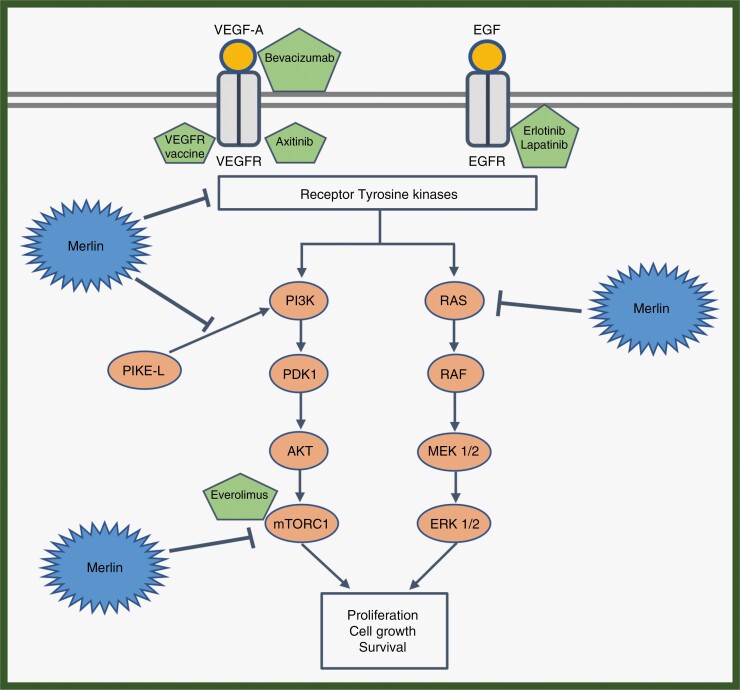
Signaling pathways regulated by Merlin are shown along with therapeutic targets and their targeted agent. Merlin regulates the activity of the receptor tyrosine kinases and inhibits their downstream signaling pathways. Merlin inhibits the PI3K-AKT-mTORC1 pathway through PIKE-L and through mTORC1. Merlin inhibits the RAS-Raf-MEK1/2 pathway as well. In this manner, it regulates cell proliferation, cell growth, and survival. In the absence of merlin in vestibular schwannomas in patients with *NF2-*related Schwannomatosis there is increased activity in these pathways leading to tumor growth. In such a situation, these therapeutic agents can act on their targets to regulate these pathways and reduce cell growth and proliferation, leading to tumor control.

By using the previously mentioned search string in PubMed and EMBASE databases, 721 results were obtained. After removing the duplicates, relevant articles were screened according to the inclusion and exclusion criteria and subsequently, they were assessed for eligibility. Finally, 13 articles were selected. An additional 3 studies were selected from citation searching and were included as well (Figure 1). In total, 16 studies were included in this review (8 prospective and 8 retrospective) investigating bevacizumab (target: VEGF-A) and 5 other targeted agents, everolimus (mTORC1 inhibitor), lapatinib (tyrosine kinase inhibitor (TKI), target: HER1 and HER2), erlotinib (TKI, target: HER1/EGFR), axitinib (TKI, target: VEGFR-1, -2 and -3) and a VEGF receptor (VEGFR) vaccine (target: VEGFR-1 and -2) (Figure 2).

### Overview of Studies Investigating Bevacizumab

A systematic search of the databases yielded 10 studies concerning bevacizumab (3 prospective and 7 retrospective) published from 2012 to 2020 including a total of 200 patients. An overview of the patient characteristics is shown in Table 1. The cohort sizes varied among the studies, from 3 patients to 61 patients with ages ranging from 10 to 79 years. Most of the studies included both adult and pediatric patients with overweight adult patients. However, 2 studies were specifically conducted with pediatric patients. A total of 51% of the participants were female. All patients were treated with bevacizumab intravenously with doses ranging from 5 mg/kg/2 weeks to 10 mg/kg/2 weeks. Additionally, some studies also included a lower maintenance dose of 2.5 mg/kg/2 weeks. The duration of treatment varied between the studies from 3 months to 76 months and in the prospective studies, the duration of treatment was usually synonymous with the follow-up period. An overview of the hearing outcomes, radiographic tumor response and toxicity can be seen in Table 2.

**Table 1.** Patient Characteristics of Studies Investigating Bevacizumab

Study	Study Design	Cohort Size	Median Age (Range)	Gender (%)	Dosage and Scheduling
Morris et al. 2016^11^	Prospective (OCS)	61	25 (10–57)	F: 25 (41%)	5 mg/kg/2 weeks or 7.5 mg/kg/3 weeks
Blakeley et al. 2016^8^	Prospective phase II	14	30 (14–79)	F: 10 (71%)	7.5 mg/kg/3 weeks
Plotkin et al. 2019^9^	Prospective, phase II	22	23 (12–62)	F: 13 (59%)	10 mg/kg/2 weeks for 6 months followed by 5 mg/kg/3 weeks
Alanin et al. 2014^17^	Retrospective	12	34 (23–78)	F: 7 (58%)	10 mg/kg/2 weeks for 6 months 15 mg/kg/3 weeks hereafter
Plotkin et al. 2012^10^	Retrospective	31	26 (12–73)	F: 17 (55%)	5 mg/kg/2 weeks
Farsctschi et al. 2015^18^	Retrospective	3	30 (28–44)	F: 1 (33%)	5 mg/kg/2–3 weeks Later reduced to 2.5 mg/kg/2–3 weeks
Goutagny et al. 2016^19^	Retrospective	16	NR	NR	5 mg/kg/2 weeks
Hochart et al. 2014^12^	Retrospective	7	15 (11–18)	F: 4 (57%)	5–10 mg/kg/2 weeks
Sverak et al. 2019^20^	Retrospective	17	30 (15–57)	F: 7 (41%)	5–10 mg/kg every 2-6 weeks
Renzi et al. 2019^13^	Retrospective	17	15 (10–17)	F: 9 (53%)	5–10 mg/kg/2–3 weeks
Pooled data		200^*^	(10–79)	F: 93 (51%)	5–10 mg/kg/2–3 weeks

^*^Not all 200 patients were tested for hearing response and radiographic tumor response.

Abbreviations: OCS, Observational clinical study; F, female; NR, not reported.

**Table 2.** Bevacizumab, Efficacy, and Toxicity

Study	Best HR (Response/ Testable)	Best HD (Response/ Testable)	Best RR (Response/ Testable)	Best PD (Response/ Testable)	Toxicity (Grade 3 or 4 AE)
Morris et al. 2016^11^	45% (5/11)	6% (2/33)	32% (15/47)	17% (8/47)	Proteinuria (2%), hypertension (2%), psychosis (2%), anemia (2%), depression (2%), infections (2%), raised alanine aminotransferase (2%)
Blakeley et al. 2016^8^	36% (5/14)	0% (0/14)	43% (6/14)	0% (0/14)	Hypertension (14%), idiopathic thrombocytopenia purpura (7%)
Plotkin et al. 2019^9^	41% (9/22)	9% (2/22)	32% (7/22)	0% (0/22)	Hypertension (25%), abdominal pain (12.5%)
Alanin et al. 2014^17^	33% (3/9)	22% (2/9)	50% (6/12)	8% (1/12)	1 death due to subarachnoid hematoma (8%)
Plotkin et al. 2012^10^	57% (13/23)	15% (4/26)	55% (17/31)	10% (3/31)	Hypertension (3%), menorrhagia (6%), irregular menstruation (6%), neutropenia (3%), vascular access complication (3%)
Farsctschi et al. 2015^18^	0% (0/3)	0% (0/3)	100% (3/3)	0% (0/3)	NR
Goutagny et al. 2016^19^	NR	NR	36% (8/22) ^*^	9% (2/22) ^*^	NR
Hochart et al. 2014^12^	25% (1/4)	NR	14% (1/7)	NR	Hypertension (14%), osteitis (14%)
Sverak et al. 2019^20^	56% (5/9)	11% (1/9)	47% (8/17)	6% (1/17)	Hypertension and proteinuria were most common
Renzi et al. 2019^13^	62% (8/13) ^**^	NR	12% (2/17)	NR	None
Pooled data (95% CI)	(49/108) 45% (36–54)	(11/116) 10% (4–15)	(73/192) 38% (32–45)	(15/168) 9% (5–13)	

^*^Target tumor not defined; result based on 22 tumors in 16 patients.

^**^Response based on results from audiogram.

Abbreviations: HR, hearing response; HD, hearing decline; RR, Radiological response; PD, progressive disease; AE, Adverse event; NR, not reported; CI, Confidence interval.

### Hearing Response to Bevacizumab

HR was measured in 9 out of the 10 studies and it varied from 0% to 62%. The pooled HR rate calculated out of 108 testable participants was 45% (95% CI: 36%–54%)

HD was measured in 7 studies and ranged from 0 to 22%. The pooled HD rate calculated out of 116 participants was 10% (95% CI: 4%–15%).

### Radiographic Tumor Response to Bevacizumab

The RR was measured in all studies, and it varied from 12% to 100%. The pooled RR rate calculated out of 192 participants was 38% (95% CI: 32%–45%).

PD was measured in 8 studies, and it ranged from 0% to 17%. Consequently, the pooled PD rate calculated out of 168 participants was 9% (95% CI: 5%–13%).

### Toxicity to Bevacizumab

Most studies reported proteinuria, hypertension, fatigue, mucositis, and menstrual abnormalities like menorrhagia and amenorrhea as the most common side-effects (CTCAE grade 1–4). However, hypertension was the most common grade 3 or 4 adverse events measured according to the CTCAE system.

### Dosage of Bevacizumab

Out of the 200 patients treated with bevacizumab, 122 patients received dosage equivalent to 2.5 mg/kg/week, 34 patients received dosage equivalent to 5 mg/kg/week, and the dosage was poorly defined for 44 patients. A comparison analysis was done to analyze the impact of bevacizumab dosage (2.5 mg/kg/week vs. 5 mg/kg/week) on HR and RR. This analysis yielded no significant results among prospective studies alone (*P* > .8) or prospective and retrospective studies combined (*P* > .4)

### Median TTP of Bevacizumab

Two of the ten studies attempted to measure TTP but failed to reach median TTP during the follow-up period of 29.3 months and 36 months, respectively.^10,19^

### Efficacy and Toxicity to Other Targeted Treatments

The systematic search yielded 6 studies investigating the efficacy and toxicity of 5 other drugs in small cohorts (7–21) with ages ranging from 10 to 63 years. An overview of these studies is shown in Table 3.

**Table 3.** Overview of Studies Investigating the Efficacy of Other Drugs

Study	Drug type and Study Design	Cohort Size	Median Age (Range)	Gender	Best Hearing Response in Target Ear (%)	Best Radiographic Tumor Response in Target Tumors (%)	Median Time to Disease Progression
Karajannis et al. 2014^15^	Everolimus, phase II	10	27 (12–44)	M: 7 (70%)	0/9 HR	0/9 RR^*^ 1/9 PD (11%)	8 months
Goutagny et al. 2015^14^	Everolimus, phase II	10	NR	M: 5 (50%)	0/9 HR 9/9 SH (100%) 0/9 HD	0/9 RR 5/9 SD (55%) 4/9 PD (44%)	> 12 months, not reached during study
Karajannis et al. 2012^16^	Lapatinib, phase II	21	28 (10–51)	M: 13 (62%)	4/13 HR (31%)	1/17 RR (6%)	14 months
Plotkin et al. 2010^21^	Erlotinib, retrospective study	11	31 (16–63)	M: 7 (64%)	1/6 HR (17%) 3/7 SH (43%) 4/7 HD (57%)	0/10 RR 4/10 SD (40%)	9.2 months
Tamura et al. 2019^22^	VEGF receptor vaccine, Phase II	7	NR (17–41)	M: 3 (43%)	2/5 (40%) HR	2/7 RR (29%) 5/7 SD (71%)	NR
Phadnis et al., 2020^23^	Axitinib, Clinical trials [NCT02129647]	12	NR (14–56)	NR	3/12 HR (25%)	2/12 RR (17%)	NR

^*^Radiological response determined as 15% reduction in volume as opposed to the other studies that define radiological response as > 20% decrease in volume.

Abbreviations: M, male; HR, hearing response; HD, hearing decline; SH, stable hearing; RR, Radiological response; PD, progressive disease; SD, stable disease; NR, not reported.

None of the studies on everolimus or erlotinib showed improvements in hearing or radiographic results.^14,15,21^

A phase II clinical trial testing the efficacy of lapatinib, showed response rates: 6% RR and 31% HR with delayed wound healing in 4.8% of participants as the only grade 3 toxicity. The median TTP was measured to be 14 months.^16^

In another phase II study, patients administered with axitinib showed hearing and radiographic tumor responses (25% HR and 17% RR). However, due to frequently observed grade 2 adverse events involving diarrhea, hematuria, skin toxicity, and grade 3 hypertension, further clinical development was recommended to be discontinued.^23^

Lastly, a phase 2 trial investigating a VEGF receptor vaccine targeting VEGFR-1 and VEGFR-2, showed response rates: 40% HR and 29% RR with no grade 3 adverse events related to treatment as evaluated by an external panel.^22^

## Discussion

In this systematic review, we included 16 studies. Bevacizumab was tested in 10 of these studies in a total of 200 patients of which the majority were adults (age range: 10–79 years). Dosage and scheduling varied across the studies (dosage range: 5–10 mg/kg/2–3 weeks).

Nevertheless, the pooled RR of 38% (95% CI: 32%–45%) and HR of 45% (95% CI: 36%–54%) did not differ significantly from the 2 prospective trials by Morris et al. and Blakeley et al. in which bevacizumab was administered at a relatively low dosage (2.5 mg/kg/week).^8,11^ Moreover, when comparing the included studies using low dosage with studies using high dosage, no significant difference in HR and RR was observed. Although solid conclusions about dosing cannot be made without a randomized trial, a recently published phase-2 trial studying low-dosage bevacizumab (5 mg/kg every 3 weeks as maintenance therapy) also showed comparable activity to higher dosages.^24^

Interestingly, grade 3 hypertension tended to be less frequent in prospective studies using lower dosages (2% and 14% vs. 25%). This could suggest that a lower dosage of bevacizumab, compared to a higher dosage, is equally effective and associated with less toxicity. However, these conclusions are not fully supported by the data provided in this review due to small cohorts and suboptimal reports of toxicity in most retrospective and prospective studies. Although, studies in more malignant brain tumors show that a lower dosage of bevacizumab is associated with lesser toxicity.^25–27^

The most serious adverse event was a brain hemorrhage involving one patient who died due to a subarachnoid hematoma. Hemorrhages are well-known side-effects of bevacizumab^28^ and therefore, bleeding “dispositions,” including usage of anti-coagulation agents and increased fall risks associated with vertigo, should be taken into account when administering bevacizumab.

In general, toxicity is not registered systematically in retrospective and prospective observational cohort studies. However, when including other available reports, hypertension, and proteinuria emerge as the most frequent bevacizumab-related toxicities leading to postponed therapy administration.^11,29,30^ Hypertension can be managed with antihypertensive treatment and most often proteinuria will normalize after a pause in treatment. However, there have been reported few patients where proteinuria may persist even after the cessation of treatment, causing long-term renal dysfunction.^29^ Therefore, blood pressure and urine analysis for protein are still recommended monitored during treatment.

Younger age has been shown to be correlated to more aggressive tumors with faster growth rates and poor response to bevacizumab therapy.^9,11,12^ This is emphasized in the study by Hochart et al involving pediatric patients with VS, which observed faster pretreatment tumor growth rates and a significantly lower RR of 14% and HR of 25% when compared to the pooled values.^12^ This poor prognosis in pediatric populations could be attributed to a more severe genotype of *NF2*-related SWN, potentially explaining the relative lack of bevacizumab efficacy.^31–33^ Despite these low response rates, most studies showed that pediatric patients had stable, progression-free disease during treatment.^9,12^ However, in future clinical trials, it could be considered to stratify for adults and pediatric patients while also investigating the correlation between age of onset, prognosis, bevacizumab efficacy, and genotypic variant.

The 6 studies testing targeted agents other than bevacizumab were small, sample-sized studies (range: 7–21 patients). Everolimus, an mTOR inhibitor, showed no HR or RR,^14,15^ which could be related to incomplete target inhibition in the tumor tissue as observed by a recent phase 0 study.^34^ Lapatinib, a TKI targeting HER1 and HER2, was well-tolerated and produced HR in 4 patients (31%) and RR in 1 patient (6%). The median TTP for lapatinib was reported to be 14 months^16^ which was less than that for bevacizumab (median TTP > 3 years^10^) but better than any other agent. Erlotinib, a TKI targeting HER1/EGFR, showed little to no activity in terms of HR and RR. Moreover, a significant portion of the cohort experienced disease progression during the treatment and consequently, no future studies have been conducted.^21^ Axitinib, another TKI targeting VEGFR-1, -2, and -3, showed HR in 3 patients and RR in 2 patients; however, due to frequent grade 2 and grade 3 toxicity, further investigation was discontinued.^23^ Finally, the VEGF receptor vaccine targeting VEGFR-1 and VEGFR-2 by Tamura et al., achieved 40% HR and 29% RR and the authors reported lower toxicity than bevacizumab treatment. Notably, the vaccine also caused tumor response in one patient, who had previously been treated with bevacizumab unsuccessfully.^22^

Median TTP was rarely measured by the studies investigating bevacizumab. In the few studies that did measure it, median TTP was prolonged (>29 months) and was not reached within the follow-up period of the study.^10,19^ In contrast, numerous studies investigating drugs other than bevacizumab found it relevant to measure median TTP, which was significantly shorter (8–14 months).^14–16,21^ Consequently, median TTP serves as a more suitable endpoint in these studies where achieving RR or HR was more challenging.

To summarize, bevacizumab showed the highest efficacy rates among all the therapies. Other targeted agents were less effective than bevacizumab. Nevertheless, lapatinib and the VEGF receptor vaccine showed some activity and had a manageable safety profile. They also slowed disease progression and stabilized tumor size and hearing. Therefore, they should be considered for future clinical trials either as monotherapy or as combination therapy. Ultimately, all tested agents included in the study targeting the classical oncogenic pathways and other drug targets are currently being tested.^5^

Lastly, this review was limited by the significant proportion of retrospective studies included. They carry with them some classical limitations like a heterogeneous cohort and nonsystematic dosage scheduling and data registration.

## Conclusion

Bevacizumab was effective in reducing the tumor size and improving the hearing in nearly 40% of patients. Compared to higher bevacizumab dosage (5 mg/kg/week), lower dosage (2.5 mg/kg/week) showed comparable efficacy and may be associated with reduced toxicity. Therefore, lower dosages of bevacizumab could be considered in the future treatment of VS in patients with *NF2-*related SWN. Other targeted agents like lapatinib and the VEGF receptor vaccine also showed some activity. However, there is still a need to improve the treatment of these patients with novel drugs and new combination therapies.
